# Prognostic significance of urokinase (uPA) and its inhibitor PAI-1 for survival in advanced ovarian carcinoma stage FIGO IIIc

**DOI:** 10.1038/sj.bjc.6690278

**Published:** 1999-04

**Authors:** W Kuhn, B Schmalfeldt, U Reuning, L Pache, U Berger, K Ulm, N Harbeck, K Späthe, P Dettmar, H Höfler, F Jänicke, M Schmitt, H Graeff

**Affiliations:** FrauenklinikInstitut für Allgemeine Pathologie und Pathologische Anatomie Institut für Medizinische Statistik und Epidemiologie, der Technischen Universität München, Klinikum rechts der Isar, München, Germany; Universitätsfrauenklinik, Klinikum Eppendorf, Hamburg, Germany

**Keywords:** prognostic factors, ovarian cancer

## Abstract

Strong evidence has accumulated on the prognostic value of tumour-associated proteolytic factors in patients afflicted with solid malignant tumours, including advanced ovarian cancer. We evaluated the prognostic impact of the protease urokinase plasminogen activator (uPA) and its inhibitor PAI-1 on overall survival in patients with advanced ovarian cancer stage FIGO IIIc in order to select patients at risk. uPA and PAI-1 antigen were determined by ELISA in primary tumour tissue extracts of 86 ovarian cancer patients FIGO stage IIIc enrolled in a prospective study. Univariate and multivariate analyses were performed using the Cox proportional hazard model. The time-varying coefficient model of Gray was used to assess the time-dependent strength of prognostic factors tumour mass, uPA and PAI-1 on overall survival. In all patients, uPA and PAI-1 (optimized cut-offs of 2.0 and 27.5 ng mg^−1^ protein respectively), in addition to the traditional prognostic parameters of residual tumour mass, nodal status, grading and ascites volume, were of prognostic significance in univariate analysis for overall survival. Even in patients with residual tumour mass (*n* = 43), the statistically independent prognostic impact of PAI-1 persisted, allowing further discrimination between low- and high-risk patients. In multivariate analysis, residual tumour mass (*P* < 0.001, relative risk (RR) 4.5), PAI-1 (*P* < 0.001; RR 3.1) and nodal status (*P* = 0.022, RR 2.6) turned out to be strong, statistically independent prognostic parameters. Evaluation of the time-dependent prognostic impact of residual tumour mass and PAI-1 on overall survival (*n* = 86, 50 months) revealed that the prognostic power of these factors increased with time. In patients with advanced ovarian cancer, both residual tumour mass and PAI-1 are statistically independent strong prognostic factors. Even within patient subgroups with or without residual tumour mass, PAI-1 allowed selection of patients at risk who might benefit from individualized therapy protocols. © 1999 Cancer Research Campaign


*British Journal of Cancer* (1999) **79**(11/12), 1746–1751
© 1999 Cancer Research Campaign
Article no. bjoc.1998.0278

### Prognostic significance of urokinase (uPA) and its inhibitor PAI-1 for survival in advanced ovarian carcinoma stage FIGO IIIc

**W Kuhn[1], B Schmalfeldt[1] U Reuning[1], L Pache[1], U Berger[3], K Ulm[3], N Harbeck,[1] K Späthe[1], P Dettmar[2], H Höfler[2], F Jänicke[4], M Schmitt[1] and H Graeff[1]**

[1]Frauenklinik, [2]Institut für Allgemeine Pathologie und Pathologische Anatomie, and[3] Institut für Medizinische Statistik und Epidemiologie, der Technischen Universität München, Klinikum rechts der Isar, München, Germany; [4]Universitätsfrauenklinik, Klinikum Eppendorf, Hamburg, Germany

**Summary** Strong evidence has accumulated on the prognostic value of tumour-associated proteolytic factors in patients afflicted with solid malignant tumours, including advanced ovarian cancer. We evaluated the prognostic impact of the protease urokinase plasminogen activator (uPA) and its inhibitor PAI-1 on overall survival in patients with advanced ovarian cancer stage FIGO IIIc in order to select patients at risk. uPA and PAI-1 antigen were determined by ELISA in primary tumour tissue extracts of 86 ovarian cancer patients FIGO stage IIIc enrolled in a prospective study. Univariate and multivariate analyses were performed using the Cox proportional hazard model. The time-varying coefficient model of Gray was used to assess the time-dependent strength of prognostic factors tumour mass, uPA and PAI-1 on overall survival. In all patients, uPA and PAI-1 (optimized cut-offs of 2.0 and 27.5 ng $mg^{-1}$ protein respectively), in addition to the traditional prognostic parameters of residual tumour mass, nodal status, grading and ascites volume, were of prognostic significance in univariate analysis for overall survival. Even in patients with residual tumour mass ($n$ = 43), the statistically independent prognostic impact of PAI-1 persisted, allowing further discrimination between low- and high-risk patients. In multivariate analysis, residual tumour mass ($P$ < 0.001, relative risk (RR) 4.5), PAI-1 ($P$ < 0.001; RR 3.1) and nodal status ($P$ = 0.022, RR 2.6) turned out to be strong, statistically independent prognostic parameters. Evaluation of the time-dependent prognostic impact of residual tumour mass and PAI-1 on overall survival ($n$ = 86, 50 months) revealed that the prognostic power of these factors increased with time. In patients with advanced ovarian cancer, both residual tumour mass and PAI-1 are statistically independent strong prognostic factors. Even within patient subgroups with or without residual tumour mass, PAI-1 allowed selection of patients at risk who might benefit from individualized therapy protocols.

**Keywords:** prognostic factors; ovarian cancer

Ovarian cancer constitutes the principal cause of death among female genital malignancies. During the last years, refined and more radical operation techniques as well as adjuvant combination chemotherapy have significantly improved the survival of patients with advanced ovarian cancer (Griffiths et al, 1979; Heintz et al, 1986; Bertelsen, 1990; Jänicke et al, 1992; Kuhn et al, 1993). Yet, radical resection of the tumour mass, which so far represents the strongest prognostic marker for survival probability, still could not prevent disease recurrence and subsequent death in over 70% of cases. In view of this, other prognostic markers are needed in order to identify patients at risk, with the aim of individualizing therapeutic strategies. Several tumour biological factors have been proposed and their prognostic strength evaluated including oestrogen, progesterone and epidermal growth factor (EGF) receptor, DNA-ploidy, S phase, and oncogenes (Friedlaender et al, 1989; Berchuck et al, 1991; Tyson et al, 1991; Lage et al, 1992). However, due to rather controversial data obtained from studies exhibiting great heterogeneity of cancer stages within the patient cohorts and often enrolling low patient numbers, so far none of these parameters has been introduced into clinical routine. Nonetheless, increasing attention has been paid to the prognostic impact of components of the plasminogen activator system conductive to extracellular matrix breakdown, thereby favouring tumour spread and metastasis (Danø et al, 1985; Schmitt et al, 1997*a*). Upon interaction of the serine protease urokinase plasminogen activater (uPA) with its receptor uPAR (CD87) on tumour cells, plasminogen is converted to the serine protease plasmin, thus focusing proteolytic activity to the cell surface (Roldan et al, 1990). Enzymatic activity of uPA is regulated by the plasminogen activator inhibitors PAI-1 and PAI-2 (Conese and Blasi, 1995). For a large series of solid cancers, uPA and PAI-1 have been established as reliable, statistically independent prognostic factors to predict recurrence-free and/or overall survival (Jänicke et al, 1989; Duffy et al, 1990; Foekens et al, 1992; Grøndahl-Hansen et al, 1993; Nekarda et al, 1994; Kobayashi et al, 1994; Hofmann et al, 1996; Schmitt et al, 1997*a*).

So far only a few studies are available on the clinical impact of uPA and PAI-1 in ovarian cancer (Kuhn et al, 1994; Schmalfeldt et al, 1995; Van der Burg et al, 1996), although it has been known for some time that ovarian cancer cells synthesize uPA and PAI-1 and express uPAR on the cell surface (Astedt and Holmberg, 1976; Casslen et al, 1994). In the ascitic fluid of ovarian cancer patients, a soluble form of uPAR has been detected and characterized (Pedersen et al, 1993). Increased uPAR in serum of ovarian cancer

*Received 24 March 1998*
*Revised 7 August 1998*
*Accepted 20 August 1998*

*Correspondence to:* Walther Kuhn, Frauenklinik der Technischen Universität München, Klinikum rechts der Isar, Ismaninger Str. 22, D-81675 München, Germany

**Table 1** Patient data for ovarian cancer FIGO IIIc ($n$ = 86)

| | |
|---|---|
| Median age (range) | 60 (20–82) years |
| Median observation time (range) | |
| All patients | 31 (1–115) months |
| Patients alive | 48 (20–115) months |
| Variable | *n* |
| Histology | |
| Serous | 61 |
| Mucinous | 5 |
| Endometroid | 3 |
| Clear cell | 3 |
| Undifferentiated | 14 |
| Grading | |
| G1 | 6 |
| G2 | 19 |
| G3 | 50 |
| G4 | 11 |
| Nodal status | |
| N0 | 24 |
| N1 | 46 |
| Nx | 16 |
| Ascites volume | |
| ≤500 ml | 51 |
| >500 ml | 35 |
| Residual tumour mass | |
| No | 43 |
| Yes | 43 |
| Lymphadenectomy | |
| Pelvic | 7 |
| Paraaortic | 4 |
| Pelvic + paraaortic | 45 |
| Sampling | 10 |
| None | 20 |
| Chemotherapy | |
| Platinum/paclitaxel | 73 |
| Other | 8 |
| None | 5 |

patients is associated with a poor prognosis (Sier et al, 1998). Elevated antigen levels of uPA and PAI-1 have been determined in invasive ovarian cancer tissue and correlated with the degree of tumour spread (Pujade-Lauraine et al, 1993). In 1994, our group was the first to conduct a systematic statistical analysis in patients with advanced ovarian cancer stage FIGO IIIc proving the prognostic value of elevated primary tumour tissue levels of uPA and PAI-1 (Kuhn et al, 1994). The prospective study presented here consolidates the strong prognostic impact of PAI-1 and residual tumour mass in an enlarged cohort of advanced ovarian cancer patients stage FIGO IIIc. We also investigate the time-dependence of the strength of these prognostic factors by applying the statistical model of Gray (1992).

### MATERIALS AND METHODS

### Patients and surgical treatment

Eighty-six patients (1986–1996) afflicted with advanced ovarian cancer stage FIGO IIIc (Fédération Internationale de Gynécologie et d'Obstétrique) were enrolled in a prospective study conducted at the Frauenklinik der Technischen Universität München (Table 1). Standard surgical procedures were performed including partial resection of the small and large intestine, diaphragmatic peritoneum, peritonectomies and upper abdominal surgery, as well as pelvic and para-aortic lymphadenectomy if indicated (Kuhn et al, 1993, 1998). The aim of the surgical procedure was to remove the entire tumour; postoperative macroscopically visible tumour was the criterion for defining the presence or absence of residual tumour. Seventy-three patients received standard chemotherapy postoperatively based on platinum and/or paclitaxel. Eight patients treated otherwise were diagnosed before 1990 and received cyclophosphamide in a palliative fashion. Five patients did not receive chemotherapy due to unfavourable health conditions. The median values of follow-up were 31 months for all and 48 months for patients still alive at time of follow-up.

### Tumour tissue collection and ELISA

Tissue samples from primary ovarian cancer ($n$ = 86) were collected during surgery, classified by the pathologist and stored in liquid nitrogen till use. Control tissue extracts were prepared from 20 benign ovarian tumors. Deep-frozen specimens of 200–500 mg wet weight were pulverized using the 'Micro-Dismembrator' (Braun, Melsungen, Germany) and immediately suspended in 2 ml Tris-buffered saline (TBS: 0.02 M Tris-HCl, pH 8.5, 125 mM sodium chloride), 1% (v/v) Triton X-100 (Sigma, München, Germany). Extraction was conducted at 4°C for 12 h followed by ultracentrifugation at 100 000 ***g*** for 45 min to separate cell debris. uPA and PAI-1 antigen concentrations were determined in the supernatant by commercially available enzyme-linked immunosorbent assay (ELISA) kits Imubind uPA #894 and Imubind PAI-1 #821 respectively (American Diagnostica, Greenwich, CT, USA) (Kuhn et al, 1994). Protein concentrations were determined by the BCA protein assay procedure (Pierce, Rockford, IL, USA). Antigen concentrations for uPA and PAI-1 are expressed as ng $mg^{-1}$ of extracted tissue protein.

### Statistical methods

Univariate and multivariate analyses were performed according to the proportional hazard model of Cox using the SPSS software package (SPSS Inc., Chicago, IL, USA). The prognostic impact was tested using the Wald test; survival probability was calculated according to Kaplan and Meier (1958). For quantitative parameters (uPA, PAI-1, age and S phase) cut-off levels were calculated by the CART (Classification And Regression Tree) method using the log-rank statistic. Cutoffs with maximal log-rank statistic were taken to discriminate between the categories 'high' and 'low' for quantitative parameters. Significance level of differences between the antigen levels in cancer tissues versus control tissues were evaluated using Mann–Whitney $U$-test. Differences were considered to be significant if $P < 0.05$. A possible time variation of the prognostic influence (relative risk) of prognostic factors was evaluated as described by Ulm et al (1997) and Schmitt et al (1997*b*), applying the statistical framework of Gray (1992). In this application of Gray's framework, Cox's proportional hazard assumption is relaxed by introducing time-dependent coefficients $\beta(t)$. Thus, the relative risk (RR) is expressed in the form:

$$RR\ (t,x) = \exp\ (\beta[t]x).$$

Analyses were performed using the statistical software program S-Plus (Statistical Sciences, 1993).

© Cancer Research Campaign 1999

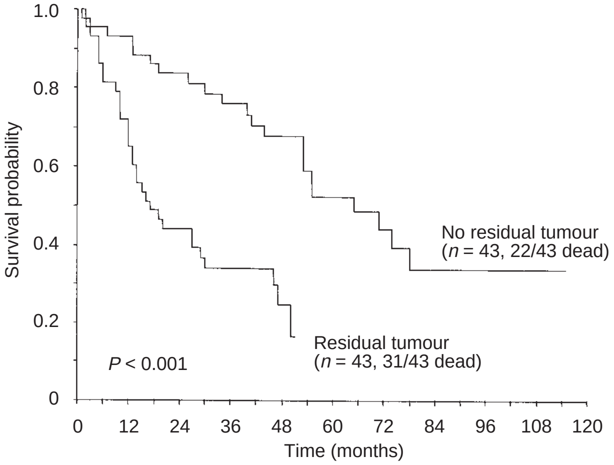

**Figure 1** Overall survival of advanced ovarian cancer patients stage FIGO IIIc ($n = 86$) as a function of residual tumour mass. Patients with no residual tumour burden ($n = 43$) had a median survival time of 65 months compared to 17 months for patients with residual tumour mass ($n = 43$). The difference between the two groups exhibited statistical significance

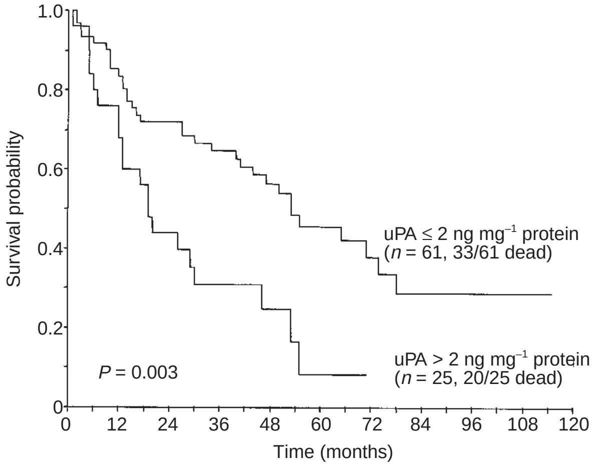

**Figure 2** Overall survival of advanced ovarian cancer patients stage FIGO IIIc ($n = 86$) as a function of uPA antigen content in primary tumour tissue extracts. Patients with uPA ≤ 2.0 ng mg$^{-1}$ protein ($n = 61$) had a median survival of 53 months compared to 19 months for patients with uPA > 2.0 ng mg$^{-1}$ protein ($n = 25$). The difference between the two groups was statistically significant

### RESULTS

### Prognostic impact of uPA, PAI-1 and established prognostic factors on overall survival uPA and PAI-1 antigen were determined by ELISA in primary tumour tissue extracts of 86 ovarian cancer patients stage FIGO IIIc; their prognostic impact was assessed by weighting with clinical prognostic factors such as age, residual tumour mass, grading, nodal status, ascites volume, DNA-ploidy and S phase. Of these factors, only residual tumour mass, nodal status, grading and ascites volume were of statistical relevance for overall survival in univariate analysis. Macroscopically tumour-free patients ($n = 43$) had a significant survival advantage (median survival time 65 months) over patients with postoperative residual tumour mass ($n = 43$) with a median survival time of 17 months (Figure 1). Likewise, node-negative patients ($n = 24$) had a significantly better prognosis than patients with node-positive ($n = 46$) or unknown nodal status ($n = 16$). Patients with low-grade tumours ($n = 25$) as well as patients with low ascites volume ($n = 51$) also had a significantly better prognosis than patients with high-grade tumours ($n = 61$, $P = 0.015$) and elevated ascites volume ($n = 35$, $P = 0.034$) respectively. In this group of patients, only age approached prognostic significance ($P = 0.053$). DNA-ploidy and S phase, a measure of cell cycle activity, were of no statistical relevance.

Both uPA (1.06 ng mg$^{-1}$ protein, range: 0.05–8.46) and PAI-1 (14.96 ng mg$^{-1}$ protein, range: 0.14–287.6) median antigen content was significantly elevated in cancer tissues compared to benign ovarian tumour tissues ($n = 20$; uPA: 0.23, range: 0.01–2.54, PAI-1: 4.5 ng mg$^{-1}$ protein, range: 1.46–15.3, $P < 0.001$) confirming our previous results obtained with a group of 45 ovarian cancer patients (Kuhn et al, 1994). Patients exhibiting uPA concentrations below the cut-off value (2.0 ng mg$^{-1}$ protein) had a statistical survival advantage ($n = 61$, 53 months) over patients exhibiting elevated uPA antigen (19 months; $n = 25$) (Figure 2). Similarly, patients with antigen levels below the PAI-1 cut-off (27.5 ng mg$^{-1}$ protein) had a median survival of 53 months ($n = 61$) compared to 19 months ($n = 25$) for patients above the PAI-1 cut-off (Figure 3). Patients with both uPA and PAI-1 antigen content ($n = 50$) below the respective cut-off values had also a statistically significant survival advantage over patients with uPA and/or PAI-1 ($n = 36$) above the respective cut-off (65 vs 20 months; $P = 0.003$). The prognostic relevant factors of uPA, PAI-1, residual tumour mass, nodal status, grading and ascites volume were weighted by Cox multivariate analysis. Residual tumour mass (RR 4.5) and PAI-1 (RR 3.1) turned out to be the strongest, statistically independent prognostic factors (Table 2) followed by the nodal status ($P = 0.022$, RR = 2.6). In another multivariate analysis, the individual factors uPA and PAI-1 were removed from the analysis, but a composite factor (‘both factors low’ vs ‘either factor high’) was included. This composite factor was also of independent statistical significance ($P = 0.004$, RR = 2.3).

### Analysis of overall survival as a function of uPA and PAI-1 in the presence and absence of residual tumour mass

In the group of patients carrying residual tumour burden, only PAI-1, but not uPA or uPA in combination with PAI-1, was of significant prognostic value ($P < 0.001$). PAI-1 antigen below the cut-off defined a group of patients ($n = 33$) with a median overall survival of 29 months, in contrast to the group with PAI-1 above the cut-off ($n = 10$) indicating a life expectancy of only 12 months. In the group of patients with no macroscopically detectable residual tumour mass ($n = 43$), both uPA and PAI-1 were of strong prognostic relevance for overall survival. Median survival time of patients with uPA below the cut-off ($n = 32$) was 74 months compared to 26 months for patients with elevated uPA ($n = 11$; $P = 0.0019$). Patients with PAI-1 concentrations below the cut-off ($n = 28$; 74 months) had a remarkable survival advantage over patients with PAI-1 above the cut-off ($n = 15$, 41 months, $P = 0.019$). The combination of uPA and PAI-1, as defined above, was also of prognostic strength ($P = 0.0049$) although it did not reach the predictive power of uPA or PAI-1 alone.

 © Cancer Research Campaign 1999

**Table 2** Univariate and multivariate analysis of prognostic factors for survival in ovarian cancer

| Variable | Univariate | | Multivariate | |
|---|---|---|---|---|
| | *P*-value | RR | *P*-value | RR |
| Residual tumour mass | | | | |
| with vs without | < 0.001 | 3.9 (2.0–7.6)[a] | < 0.001 | 4.5 (2.2–9.3) |
| PAI-1 | | | | |
| ≤ 27.5 vs > 27.5 ng mg$^{-1}$ protein | 0.012 | 2.1 (1.2–3.6) | < 0.001 | 3.1 (1.7–5.7) |
| uPA | | | | |
| ≤ 2.0 vs > 2.0 ng mg$^{-1}$ protein | 0.003 | 2.4 1.4–4.3 | n.s. | |
| Nodal status | | | | |
| N0 vs N1, Nx | < 0.001 | 3.8 (1.7–8.2) | 0.022 | 2.6 (1.1–5.7) |
| Age | | | | |
| ≤ 60 vs > 60 years | 0.053 | – | n.s. | |
| Grading | | | | |
| G1+G2 vs G3+G4 | 0.015 | 2.4 (1.2–4.8) | n.s. | |
| Ascites volume | | | | |
| ≤ 500 vs > 500 ml | 0.034 | 1.8 (1.0–3.2) | n.s. | |

n.s. = not significant. [a]confidence interval.

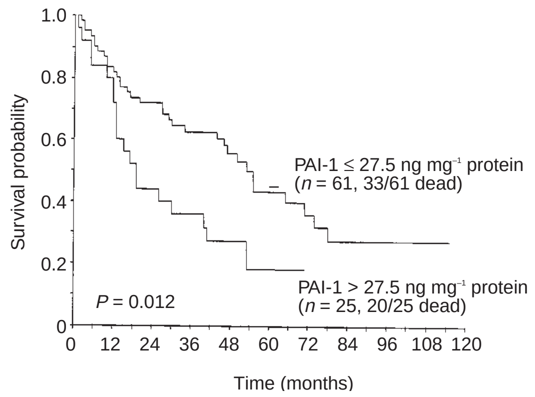

**Figure 3** Overall survival of advanced ovarian cancer patients stage FIGO IIIc ($n$ = 86) as a function of PAI-1 antigen content in primary tumour tissue extracts. Patients with PAI-1 ≤ 27.5 ng mg$^{-1}$ ($n$ = 61) had a median survival of 53 months compared to 19 months for patients with PAI-1 > 27.5 ng mg$^{-1}$ ($n$ = 25). The difference between the two groups exhibited statistical significance

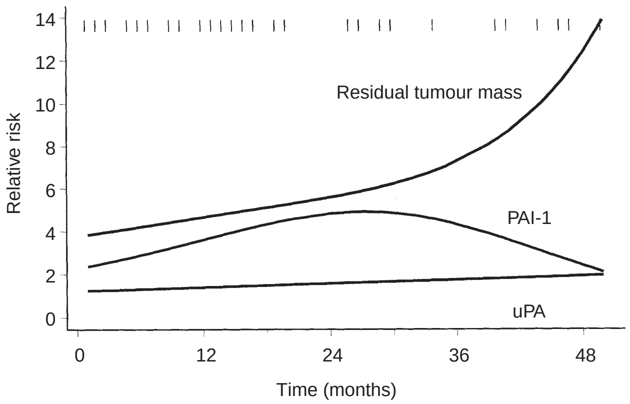

**Figure 4** Time variation of the relative risk related to uPA, PAI-1 and residual tumour mass. Statistical evaluation of the relative risks of death was conducted within a time frame of 50 months by using the time-varying coefficient model of Gray (1992)

### Time-varying prognostic impact of uPA, PAI-1 and residual tumour mass

The statistical model of Gray (1992) was applied to describe the change of the relative risk values for uPA, PAI-1 and residual tumour mass over time (50 months). This type of analysis disclosed that the prognostic influence of residual tumour mass and PAI-1 considerably and constantly increased over time, with PAI-1 reaching its peak at 2 years follow-up. There was almost no change to the relatively low prognostic power of uPA over time (Figure 4).

### DISCUSSION

Several studies have been undertaken to identify and characterize tumour biological factors in order to predict overall survival in advanced ovarian cancer (Friedlaender et al, 1989; Berchuck et al, 1991; Tyson et al, 1991; Lage et al, 1992). So far none of these factors explored was strong enough to allow translation into clinical practice, thus leaving clinicians with the major traditional prognostic factor residual tumour mass (Kuhn et al, 1993; Makar et al, 1995). In the past, the course of the disease has been improved by more radical and refined surgical techniques in combination with platinum- and paclitaxel-based chemotherapy; however, even patients without residual tumour mass often experience unsatisfactory early disease recurrence. In a recent study by Makar et al, the clinical value of residual tumour mass in predicting overall survival was consolidated for FIGO III patients, consistent with our previous data (Kuhn et al, 1993; Makar et al, 1995).

A major new finding in our present analysis ($n$ = 86) points to the strong prognostic impact of the protease inhibitor PAI-1 in FIGO IIIc patients with or without residual tumour mass. This

© Cancer Research Campaign 1999

finding allows better selection of at risk patients in these clinically important ovarian cancer subgroups according to elevated tumour antigen level of PAI-1. In a variety of human solid cancers, special attention has been paid to tumour-associated proteolytic factors crucially implicated in tumour invasion and metastasis, such as uPA and its inhibitor PAI-1 (Jänicke et al, 1989; Duffy et al, 1990; Foekens et al, 1992; Grondahl-Hansen et al, 1993; Kuhn et al, 1994; Kobayashi et al, 1994; Nekarda et al, 1994; Hofmann et al, 1996). With this knowledge in mind, our group was the first to find significantly elevated antigen levels of uPA and PAI-1 in breast cancer tissue (Jänicke et al, 1989, 1991) to be associated with disease recurrence or early death. Later it was shown that elevated levels of these factors in cancer tissue of the gastrointestinal tract, kidney, bladder, lung, brain, breast, cervix and ovary also indicate a poor prognosis (Schmitt et al, 1997*b*).

This paper presents strong evidence that, in addition to residual tumour mass, PAI-1 is a statistically independent prognostic factor in FIGO IIIc patients (Kuhn et al, 1994). Even more important, PAI-1 remains a strong, statistically relevant prognostic factor in the subgroup of patients with residual tumour mass, thus allowing discrimination of low- and high-risk patients. Despite the presence of residual tumour mass, low PAI-1 antigen defined a favourable group of patients with considerable survival advantage of more than 17 months over patients with high PAI-1 values.

In the group of patients with no macroscopically detectable residual tumour mass, both uPA and PAI-1 were of strong prognostic relevance for overall survival. Similar to the patients with residual tumour mass, elevated levels of PAI-1 and uPA allow one to define patients who have a favourable prognosis with a survival advantage of 33 and 48 months respectively. Van der Burg et al (1996), who assessed a smaller number of FIGO III patients (eight without and 36 with residual tumour mass), did not detect such an effect. Aside from the smaller collective, a major difference in the statistical methods applied may have been responsible for this difference: to identify patients at risk, we used optimized cut-off values (log-rank statistic) for uPA and PAI-1, whereas van der Burg et al employed median values. The strong clinical impact of uPA and PAI-1 in residual tumour-free patients has also been shown in totally resected ($R_o$) gastric cancer patients by our group (Nekarda et al, 1994). The statistical significance of uPA and PAI-1 for tumour-free patients in this type of cancer is similar to the situation in tumour-free ovarian cancer.

For some types of cancer (Merkel and McGuire, 1990; Dettmar et al, 1997), DNA ploidy and S phase fraction have been proposed as prognostic markers. In our group of ovarian cancer patients, none of these DNA parameters attained statistical significance, a finding supported by the data of Kigawa et al (1993) and Kaern et al (1994). On the other hand, we have shown that nuclear grade, ascites volume and nodal status are of prognostic relevance in FIGO IIIc patients, thus confirming data obtained by other study groups (Burghardt et al, 1991; Scarabelli et al, 1995).

As noted above, a statistical approach, first described by Gray in 1992, has been applied in the present analysis to calculate the change of the relative risk for early death over time (50 months) associated with presence of residual tumour mass or elevated levels of uPA or PAI-1. This type of analysis discloses that the prognostic influence of residual tumour mass and PAI-1 increase considerably over time, whereas that of uPA is not subject to change. Our group previously applied Gray's model to study the time-varying impact of uPA and PAI-1 on prognosis (Schmitt et al, 1997*a*) in breast cancer. Similar to our new findings in ovarian cancer stage FIGO IIIc, the prognostic impact of PAI-1 in breast cancer increases over time, whereas that of uPA is significant for the first 3 years only.

It is a common observation in solid malignant tumours that elevated levels of uPA and its inhibitor PAI-1 indicate poor outcome in cancer patients (Schmitt et al, 1997*b*). This clinical observation is supported by tumour biological findings pointing to a special role of PAI-1 in tumour invasion and metastasis, quite apart from its function in inhibiting the proteolytic activity of the protease uPA (Kanse et al, 1996; Stefansson and Lawrence, 1996; Wei et al, 1996). PAI-1 modulates binding of uPAR (the receptor for uPA, CD87) to vitronectin (Kanse et al, 1996) and binding of vitronectin to the cellular integrin $\alpha_v\beta_3$ (Stefansson and Lawrence, 1996) thereby facilitating tumour cell migration and metastasis. Along these lines, Liu et al (1995) have shown that co-expression of uPA, PAI-1 and uPAR by tumour cells is necessary for focalized and optimal invasion. Increases in PAI-1 are also associated with angiogenesis (Barbareschi et al, 1995).

Selection of ovarian cancer FIGO IIIc patients in the subgroups with or without residual tumour mass by the tumour biological factors uPA and/or PAI-1 could lead to individualized therapeutic regimens for patients at risk, thereby increasing the chance to improve the course of the disease and predict therapy response, especially for high-risk patients with residual tumour mass. New therapeutical protocols that may be considered for these patients suggest high-dose chemotherapy in combination with stem cell support or secondary debulking procedures (Benedetti et al, 1995; Van der Burg et al, 1995). Prior to transfer into clinical routine, further validation of the impact of uPA and PAI-1 in ovarian cancer prognosis is needed, which will require assessing larger, and different, cancer patient groups. The predictive value should then be determined by evaluating the therapy response of patients at risk in a randomized trial.

### ACKNOWLEDGEMENTS

This study was supported by the Deutsche Forschungsgemeinschaft (Klinische Forschergruppe GR 280/4–5). The generous support of Dr Richard Hart, American Diagnostica, Greenwich, CT, is highly acknowledged. We are thankful for expert technical assistance of Mrs Erika Sedlaczek and Mrs Brigitte Jaud-Münch.

### REFERENCES

Astedt B, Holmberg L (1976) Immunological identity of urokinase and ovarian carcinoma plasminogen activator released in tissue culture. *Nature* **261**: 595–597

Barbareschi M, Gasparini G, Morelli L, Forti S and Della-Palma P (1995) Novel methods for the determination of the angiogenic activity of human tumors. *Breast Cancer Res Treat* **36**: 181–192

Benedetti PP, Greggi S, Scambia G, Salerno MG, Baiocchi G, Laurelli G, Menichella G, Pierelli L, Foddai ML and Serfaini R (1995) Very high-dose chemotherapy with autologous peripheral stem cell support in advanced ovarian cancer. *Eur J Cancer* **31A**: 1987–1992

Bertelsen K (1990) Tumor reduction surgery and long-term survival in advanced ovarian cancer: A DACOVA study. *Gynecol Oncol* **38**: 203–209

Berchuck A, Rodriguez GC, Kamel A, Dodge RK, Soper JT, Clarke-Pearson DL and Bast RC Jr (1991) Epidermal growth factor receptor expression in normal ovarian epithelium and ovarian cancer. *Am J Obstet Gynecol* **164**: 669–674

Burghardt E, Girardi F, Lahousen M, Tamussino K and Stettner H (1991) Patterns of pelvic and paraaortic lymph node involvement in ovarian cancer. *Gynecol Oncol* **40**: 103–106

Casslen B, Bossmar T, Lecander I and Astedt B (1994) Plasminogen activators and plasminogen activator inhibitors in blood and tumor fluids of patients with ovarian cancer. *Eur J Cancer* **30**: 1302–1311

 *© Cancer Research Campaign 1999*

Conese M and Blasi F (1995) Urokinase/urokinase receptor system: Internalization/ degradation of urokinase-serpin complexes: mechanism and regulation. *Biol Chem Hoppe-Seyler* **376**: 143–155

Danø K, Andreasen PA, Grondahl-Hansen J, Kristensen P, Nielsen LS and Skriver L (1985) Plasminogen activators, tissue degradation and cancer. *Adv Cancer Res* **44**: 139–266

Dettmar P, Harbeck N, Thomssen C, Pache L, Ziffer P, Fizi K, Jänicke F, Nathrath W, Schmitt M, Graeff H and Höfler H (1997) Prognostic impact of proliferation-associated factors MIBl (Ki-67) and S-phase in node-negative breast cancer. *Br J Cancer* **75**: 1525–1533

Duffy MJ, Reilley D, O'Sullivan C, O'Higgins N, Fennelly JJ and Andreasen P (1990) Urokinase-plasminogen activator, a new and independent prognostic marker in breast cancer. *Cancer Res* **50**: 6827–6829

Foekens JA, Schmitt M, van Putten WLJ, Peters HA, Bontenbal M, Jänicke F and Klijn JG (1992) Prognostic value of urokinase-type plasminogen activator in 671 primary breast cancer patients. *Cancer Res* **52**: 6101–6105

Friedlander ML, Quinn MA, Fortune D, Foo MS, Toppila M, Hudson CN and Russell P (1989) The relationship of steroid receptor expression to nuclear DNA distribution and clinicopathological characteristics in epithelial ovarian tumors. *Gynecol Oncol* **32**: 184–190

Gray RJ (1992) Flexible methods for analyzing survival data using splines, with application to breast cancer prognosis. *J Am Stat Assoc* **87**: 942–951

Griffiths CT, Parker LM and Fuller AF Jr (1979) Role of cytoreductive surgical treatment in the management of advanced ovarian cancer. *Cancer Treat Reports* **63**: 235–240

Grøndahl-Hansen J, Christensen IJ, Rosenquist C, Brünner N, Mouridsen HT, Danø K and Blichert-Toft M (1993) High levels of urokinase-type plasminogen activator (uPA) and its inhibitor PAI-1 in cytosolic extracts of breast carcinomas are associated with poor prognosis. *Cancer Res* **53**: 2513–2521

Heintz APM, Hacker NF, Berek JS, Rose TP, Munoz AK and Lagasse LD (1986) Cytoreductive surgery in ovarian carcinoma: feasibility and morbidity. *Obstet Gynecol* **67**: 783–788

Hofmann R, Lehmer A, Buresch M, Hartung R and Ulm K (1996) Clinical relevance of urokinase plasminogen activator, its receptor, and its inhibitor in patients with renal cell carcinoma. *Cancer* **78**: 487–492

Jänicke F, Schmitt M, Ulm K, Gössner W and Graeff H (1989) Urokinase-type plasminogen activator antigen and early relapse in breast cancer. *Lancet* **8670**: 1049

Jänicke F, Hölscher M, Kuhn W, von Hugo R, Pache L, Siewert JR and Graeff H (1992) Radical surgical procedure improves survival time in patients with recurrent ovarian cancer. *Cancer* **70**: 2129–3216

Kaern J, Trope CG, Kristensen GB and Pettersen EO (1994) Flow cytometric DNA ploidy and S-phase heterogeneity in advanced ovarian carcinoma. *Cancer* **73**: 1870–1877

Kanse S, Kost C, Wilhelm O, Andreasen P and Preissner K (1996) The urokinase receptor is a major vitronectin-binding protein on endothelial cells. *Exp Cell Res* **224**: 344–353

Kaplan EL and Meier P (1958) Nonparametric estimation from incomplete observations. *J Am Stat Assoc* **53**: 457–481

Kigawa J, Minagawa Y, Ishihara H, Kanamori Y and Terakawa N (1993) Tumor DNA ploidy of patients with serous cystadenocarcinoma of the ovary. *Cancer* **72**: 804–808

Kobayashi H, Fujishiro S and Terao T (1994) Impact of urokinase-type plasminogen activator and its inhibitor type-1 on prognosis in cervical cancer of the uterus. *Cancer Res* **54**: 6539–6548

Kuhn W, Jänicke F, Pache L, Hölscher M, Schattenmann G, Schmalfeldt B, Anderl H, Schüle G, Dettmar P, Siewert JR and Graeff H (1993) Entwicklungen in der Therapie des fortgeschrittenen Ovarialkarzinoms FIGO III. *Geburtshilf Frauenheilk* **53**: 293–302

Kuhn W, Pache L, Schmalfeldt B, Dettmar P, Schmitt M, Jänicke F and Graeff H (1994) Urokinase (uPA) and PAI-1 predict survival in advanced ovarian cancer patients (FIGO III) after radical surgery and platinum-based chemotherapy. *Gynecol Oncol* **55**: 401–409

Kuhn W, Florack G, Roder J, Schmalfeldt B, Pache L, Rust M, Ulm K, Späthe K, Jänicke F, Siewert JR and Graeff H (1998) The influence of upper abdominal surgery on perioperative morbidity and mortality in patients with advanced ovarian cancer FIGO III and FIGO IV. *Int J Gynecol Cancer* **8**: 56–63

Lage JM, Weinberg DS, Huettner PC, Mark SD (1992) Flow cytometric analysis of nuclear DNA content in ovarian tumors. *Cancer* **69**: 2668–2675

Liu G, Shuman MA and Cohen RL (1995) Co-expression of urokinase, urokinase receptor and PAI-1 is necessary for optimum invasiveness of cultured lung cancer cells. *Int J Cancer* **60**: 501–506

Makar AP, Baekelandt M, Trope CG and Kristensen GB (1995) The prognostic significance of residual disease, FIGO substage, tumor histology, and grade in patients with FIGO stage III ovarian cancer. *Gynecol Oncol* **56**: 175–180

Merkel DE and McGuire WL (1990) Ploidy, proliferative activity and prognosis. *Cancer* **65**: 1194–1205

Nekarda H, Siewert JR, Schmitt M, Roder, JD, Fink U, Ulm K, Becker K and Höfler H (1994) Tumour-associated proteolytic factors uPA and PAI-1 and survival in totally resected gastric cancer. *Lancet* **343**: 117

Pedersen N, Schmitt M, Rønne E, Nicoletti MI, Hoyer-Hansen G, Conese M, Giavazzi R, Dano K, Kuhn W and Jänicke F (1993) A ligand-free, soluble urokinase receptor present in ascitic fluid from patients with ovarian cancer. *J Clin Invest* **92**: 2160–2167

Pujade-Lauraine E, Lu H, Mirshahi S, Mirshahi S, Soria J, Soria C, Bernadou A, Kruithof EK, Lijnen HR and Burtin P (1993) The plasminogen-activation system in ovarian tumors. *Int J Cancer* **55**: 27–31

Røldan AL, Cubellis MV, Masucci MT, Behrendt N, Lund LR, Dano K, Appella E and Blasi F (1990) Cloning and expression of the receptor for urokinase-type plasminogen activator, a central molecule in cell-surface plasmin-dependent proteolysis. *EMBO J* **9**: 467–474

Scarabelli C, Gallo A, Zarrelli A, Visentin C and Campagnutta E (1995) Systemic pelvic and para-aortic lymphadenectomy during cytoreductive surgery in advanced ovarian cancer: Potential benefit on survival. *Gynecol Oncol* **56**: 328–337

Schmalfeldt B, Kuhn W, Reuning U, Dettmar P, Schmitt M, Jänicke F, Höfler H and Graeff H (1995) Primary tumor and metastasis in ovarian cancer differ in their content of urokinase-type plasminogen activator (uPA), its receptor (uPAR) and inhibitors type-1 (PAI-1) and type-2 (PAI-2). *Cancer Res* **55**: 3958–3963

Schmitt M, Harbeck N, Thomssen C, Wilhelm O, Magdolen V, Reuning U, Ulm K, Höfler H, Jänicke F and Graeff H (1997*a*) Clinical impact of the plasminogen activation system in tumor invasion and metastasis: prognostic relevance and target for therapy. *Thromb Hemost* **78**: 285–296

Schmitt M, Thomssen C, Ulm K, Seiderer A, Harbeck N, Höfler H, Jänicke F and Graeff H (1997*b*) Time-varying prognostic impact of tumor biological factors urokinase (uPA), PAI-1, and steroid hormone receptor status in primary breast cancer. *Br J Cancer* **76**: 306–311

Sier CM, Vloedgraven HJM, Ganesh S, Griffioen G, Quax PH, Verheijen JH, Dooijewaard G, Welvaart K, van de Velde CJ and Lamers CG (1994) Inactive urokinase and increased levels of its inhibitor type 1 in colorectal cancer liver metastasis. *Gastroenterology* **107**: 1449–1456

Sier CM, Stephens R, Biziki J, Mariani A, Bassan M, Pedersen N, Frigerio L, Ferrari A, Danø K, Brünner N and Blasi F (1998) The level of urokinase-type plasminogen activator receptor is increased in serum of ovarian cancer patients. *Cancer Res* **58**: 1843–1849

Stefansson S and Lawrence DA (1996) The serpin PAI-1 inhibits cell migration by blocking integrin alpha(v)beta(3) binding to vitronectin. *Nature* **383**: 441–443

Tyson FL, Boyer CM, Kaufman R, O'Briant K, Cram G, Crews JR, Soper JT, Daly L, Fowler WC Jr and Haskill JS (1991) Expression and amplification of the HER2/neu (c-erbB2) protooncogene in epithelial ovarian tumors and cell lines. *Am J Obstet Gynecol* **165**: 640–646

Ulm K, Dannegger F, Klinger A and Jänicke F (1997) Zeitveränderliche Effekte prognostischer Faktoren am Beispiel des Mammakarzinoms. In *Medizinische Informatik, Biometrie und Epidemiologie*, Baur MP, Fimmers R and Blettner M (eds), pp. 447–450. MMV Verlag, München

Van der Burg MEL, van Lent M, Buyse R, Kobierska A, Colombo N, Favalli G, Lacave AJ, Nardi M, Renard J and Pecorelli S (1995) The effect of debulking surgery after combination chemotherapy on the prognosis in advanced epithelial ovarian cancer. *N Engl J Med* **332**: 629–634

Van der Burg MEL, Henzen-Logmans SC, Berns EMJJ, van Putten WL, Klijn JG and Foekens JA (1996) Expression of urokinase-type plasminogen activator (uPA) and its inhibitor PAI-1 in benign, borderline, malignant primary and metastatic ovarian tumors. *Int J Cancer* **69**: 475–479

Wei Y, Lukashev M, Simon DI, Bodary SC, Rosenberg S, Doyle MV and Chapman HA (1996) Regulation of integrin function by the urokinase receptor. *Science* **273**: 1551–1555

© Cancer Research Campaign 1999
